# Temporal Dynamics of Reproductive Activity and Sexual Group Maturity of *Octopus minor* in the Deukryang Bay, Southern Coast of Korea

**DOI:** 10.3390/ani16203267

**Published:** 2026-10-16

**Authors:** Seong Jin Kim, Hyeon Jin Kim, So Ryung Shin, So Hui Kim, Young Guk Jin, Jung Sick Lee

**Affiliations:** 1 Department of Aqualife Medicine, Chonnam National University, Yeosu 59626, Republic of Korea; 2 Leading Research Center of Total Solution for Osteoporosis Treatment, Chonnam National University, Yeosu 59626, Republic of Korea; 3 Aquaculture Research Division, National Institute of Fisheries Science, Busan 46083, Republic of Korea; 4 South Sea Fisheries Research Institute, National Institute of Fisheries Science, Yeosu 59780, Republic of Korea

**Keywords:** *Octopus minor*, sex ratio, reproductive activity, spawning pattern, sexual group maturity

## Abstract

This study was performed to obtain basic information for the resource management and conservation of *Octopus minor*, one of the important fishery resources in Korea. *O. minor* used in this study were collected monthly from May 2022 to August 2023 using a drum-shaped net in Deukryang Bay, southern coast of Korea. The overall sex ratio (female:male) of *O. minor* was 1:1.03 (*n* = 322; females = 159, males = 163), with a significantly higher proportion of males at a total weight of 150.1 g and above (*p* < 0.05). The gonadosomatic index (GSI) of females reached its highest value in July, whereas the reproductive complex index (RCI) showed its lowest value. In males, the RCI reached its highest values in June and July, showing a variation pattern opposite to that of the GSI. Although the timing of changes in GSI, RCI, and gonadal morphology and histology differed between females and males, both sexes exhibited clear seasonal patterns. The main mating and spawning season was estimated as summer (July–August). The oocyte development pattern was synchronous, while the spawning pattern was monocyclic with strict seasonality. The size at 50% sexual group maturity of *O. minor* was estimated at a total weight and mantle length of 49.5 g and 43.6 mm in females, and 49.7 g and 41.9 mm in males. The information on the main spawning season and size at 50% sexual group maturity obtained from the *O. minor* population in Deukryang Bay, Korea, can be used to establish a prohibited fishing season and size limit for resource management.

## Simple Summary

In this study, the reproductive biology of *Octopus minor* collected from Deukryang Bay, southern coast of Korea, was investigated through anatomical and histological analyses of the gonads and reproductive complexes. The reproductive activity showed clear seasonal variation, and the main mating and spawning season was estimated to be summer (July–August). Analysis of oocyte development and reproductive pattern indicated that *O. minor* has a reproductive strategy characterized by a single spawning period during a specific season. The size at which 50% of individuals reached sexual maturity was 49.5 g and 49.7 g in total weight and 43.6 mm and 41.9 mm in mantle length for females and males, respectively. These results provide useful biological information for establishing appropriate fishing restrictions, including prohibited fishing seasons and size limits, for the sustainable management of *O. minor* populations in Korea.

## 1. Introduction

Octopods are widely distributed from low latitudes to polar regions and inhabit diverse environments, ranging from intertidal zones to depths exceeding 5000 m, depending on the species, and represent important fishery resources worldwide [1,2,3]. *Octopus minor* is widely distributed in the coastal waters of Korea, China, and Japan and is a benthic species that inhabits coastal waters, particularly tidal flats with muddy or sandy-mud substrates [4,5]. In Korea, *O. minor* is one of the major cephalopod fishery resources, along with *O. vulgaris* and *Todarodes pacificus*. It is particularly important to coastal fisheries in southern coast of Korea, where approximately 60% of its total domestic production occurs [6]. Its annual production peaked at 14,351 tons in 1993 and has since shown a declining trend, decreasing to 7658 tons in 2005, 6954 tons in 2010, 6690 tons in 2015, 6071 tons in 2020, and 5899 tons in 2024 [7]. This long-term decline in production highlights the need for continued management and conservation of *O. minor* populations in Korea.

Research on reproductive biology provides fundamental information for understanding the reproductive characteristics and strategies of a species, as well as for fisheries resource management and the development of aquaculture technologies. Cephalopods exhibit distinctive reproductive characteristics compared with many other marine animals. In many marine animals, including teleosts, males produce sperm in the testis and release it as a sperm suspension [8,9,10]. In contrast, sperm produced in the testis of cephalopods are packaged into spermatophores within the reproductive complex (accessory reproductive organs excluding the gonad) before being transferred to females [11,12]. Therefore, examination of the reproductive complex, together with the gonads, is important for understanding the reproductive biology and reproductive strategies of cephalopods.

Many studies have been conducted on the reproductive biology of cephalopods, including the main spawning season and reproductive cycle, in species such as *O. vulgaris* [13,14,15,16], *O. maya* [17,18], *O. hubbsorum* [19,20], and *O. minor* [21,22,23,24]. However, few studies have analyzed both the gonads and reproductive complexes of these species.

Anatomical and histological methods have been widely used in studies of reproductive biology across various marine animals, including mollusks [14,15,25,26] and teleosts [10,27]. However, even within the same species, results may differ among studies depending on research methods and geographical differences among the populations examined. Geographical differences may reflect differences in local environmental conditions that influence reproductive characteristics. Furthermore, differences in the methods used to classify gonadal developmental stages may affect the interpretation of reproductive status. Therefore, caution is needed when comparing results among studies [10,28,29,30].

In this study, anatomical and histological analysis of the gonad and reproductive complex of *O. minor* were conducted to evaluate reproductive biological indicators, including sex ratio, gonadosomatic index (GSI), reproductive complex index (RCI), and sexual group maturity. The results were compared with those of previous studies, thereby providing reliable information for resource management and conservation.

## 2. Materials and Methods

### 2.1. Sampling

**Figure 1:**
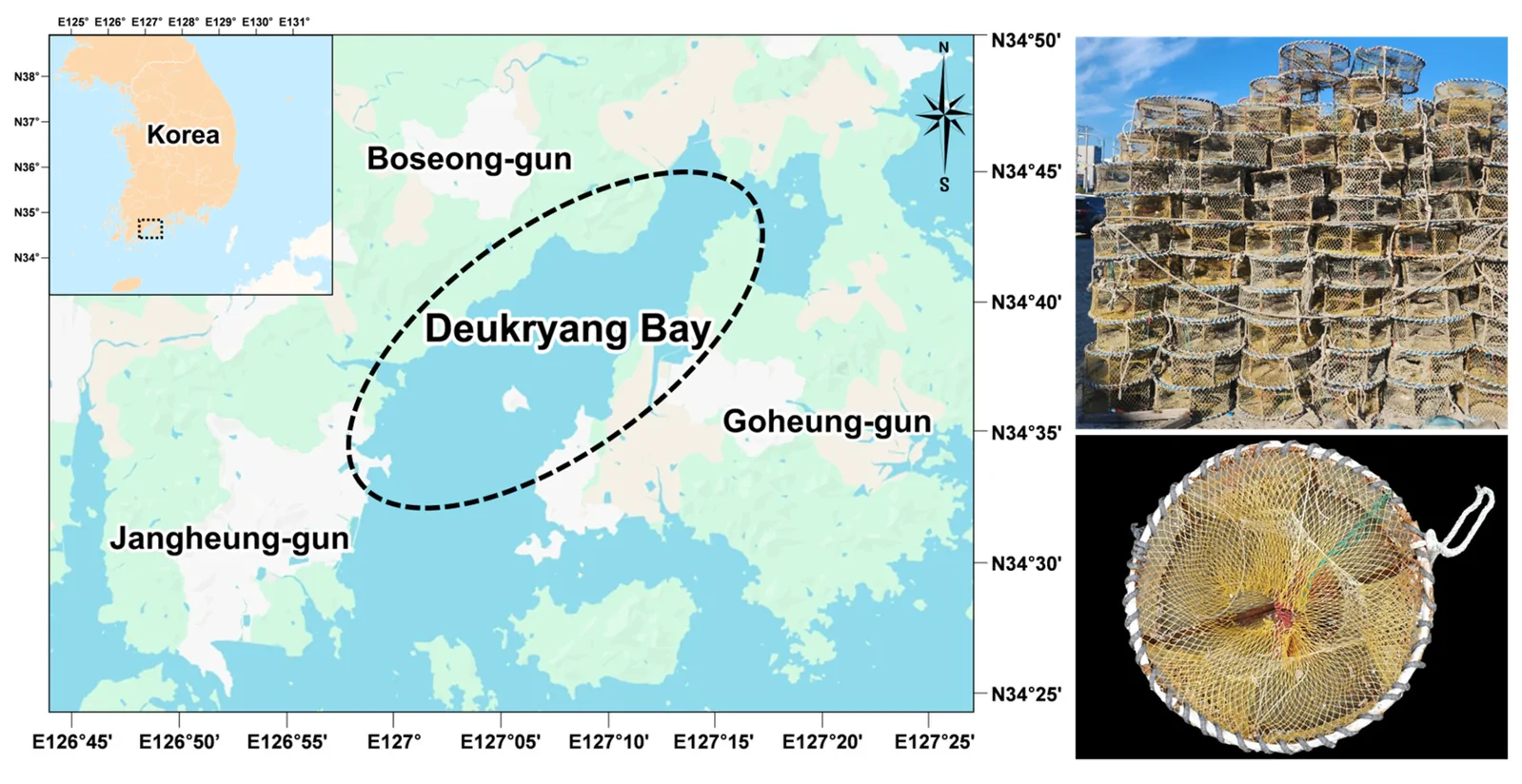
Sampling area and drum-shaped net of *Octopus minor* (Base map: Google Maps. Map data © Google).

*Octopus minor* used in this study were collected monthly from May 2022 to August 2023 using a drum-shaped net in Deukryang Bay, southern coast of Korea (Figure 1). Approximately 15–25 specimens were collected each month. A total of 322 specimens (mean total weight = 113.6 ± 70.6 g; total length = 656.7 ± 149.9 mm; mantle length = 66.3 ± 21.8 mm) were used for the analysis.

### 2.2. Sex and Sex Ratio

**Figure 2:**
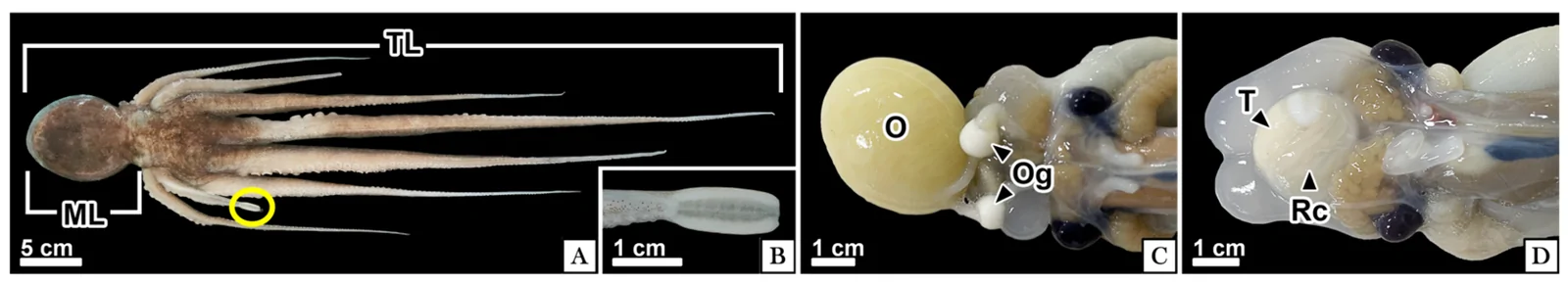
Morphology and anatomy of *Octopus minor*. (**A**) Morphometric characteristics, (**B**) hectocotylized arm (yellow circle), (**C**,**D**) reproductive organs of female (**C**) and male (**D**). ML: mantle length, O: ovary, Og: oviducal gland, Rc: reproductive complex, T: testis, TL: total length.

Sex differentiation was based on the presence of a hectocotylized arm, morphology of the gonad, and histological analysis according to the study by Kim et al. [31] (Figure 2). The sex ratio (female:male) and percentage of females in the population were calculated with the following equations.

Sex ratio = Female (*n*): Male (*n*) (1)

Female (%) = Female (*n*) × 100/[Female (*n*) + Male (*n*)] (2)

### 2.3. Gonadosomatic Index (GSI)

The GSI was calculated using the following equations and presented as the median and average values.

Female GSI = Ovary weight (g) × 100/Body weight (g)(3)

Male GSI = Testis weight (g) × 100/Body weight (g)(4)

### 2.4. Reproductive Complex Index (RCI)

The RCI was calculated using the following equations based on the methods of Hayashi [32] and Kim [33] and presented as the median and average values. The weight of the male reproductive complex was the sum of the weights of the primary vas deferens, primary spermatophoric gland, secondary spermatophoric gland, and spermatophoric sac, based on the method of Kim [33].

(5) $$\mathrm{Female RCI}=\frac{\mathrm{Oviducal gland weight}\;(g)}{\mathrm{Oviducal gland weight}\;(g)+\mathrm{Ovary weight}\;(g)}\times 100$$

(6) $$\mathrm{Male RCI}=\frac{\mathrm{Reproductive complex weight}\;(g)}{\mathrm{Reproductive complex weight}\;(g)+\mathrm{Testis weight}\;(g)}\times 100$$

### 2.5. Histological Analysis

The gonads of all specimens collected in this study were subjected to histological analysis. The samples were fixed in aqueous 10% neutral formalin for 24 h, then rinsed in running tap water for 36–48 h. Subsequently, the samples were dehydrated through a graded ethanol series (70–100%) and then embedded in paraplast (Leica, Nussloch, Germany) for 4 h. Embedded tissues were sectioned at a thickness of 4–5 μm using a microtome (RM2235, Leica, Nussloch, Germany). Samples were stained with Mayer’s hematoxylin-0.5% eosin (H-E) stain.

### 2.6. Image Analysis

Histological quantification of germ cells was performed by analyzing 15 cells at each stage. Microscopic images were converted into JPEG files and analyzed using an image analyzer (i-Solution, IMT i-solution Inc., Burnaby, BC, Canada). The size of the cells was measured as the major axis and the minor axis. The lengths of the major and minor axes of each germ cell were measured at least three times.

### 2.7. Gonadal Development Stage

The oocyte developmental stage was classified into seven stages based on size, stainability, follicle layer development, and yolk accumulation: oogonium, previtellogenic oocyte, initial vitellogenic oocyte, early active vitellogenic oocyte, late active vitellogenic oocyte, mature oocyte, and ripe oocyte [33,34].

The gonadal developmental stage was classified into five stages based on morphological and histological analyses: inactive stage, early active stage, late active and mature stage, ripe stage, and spent and degenerative stage, and these stages were compared. Morphological classification was based on the shape, size, and color.

Histological classification was based on the dominant developmental stages of germ cells, referring to the methods of Cuccu et al. [16], Kim et al. [22], and Kim [33].

In females, the inactive stage was characterized by the dominance of oogonia, previtellogenic oocytes, and initial vitellogenic oocytes; the early active stage by early active vitellogenic oocytes; the late active and mature stage by late active vitellogenic oocytes and mature oocytes; and the ripe stage by ripe oocytes. The spent and degenerative stage was characterized by the presence of degenerative oocytes.

In males, the inactive stage was characterized by the dominance of spermatogonia; the early active stage by spermatocytes; the late active and mature stage by spermatids; and the ripe stage by sperm. The spent and degenerative stage was characterized by irregularly shaped seminiferous tubules.

### 2.8. Frequency of Male with Spermatophore

**Figure 3:**
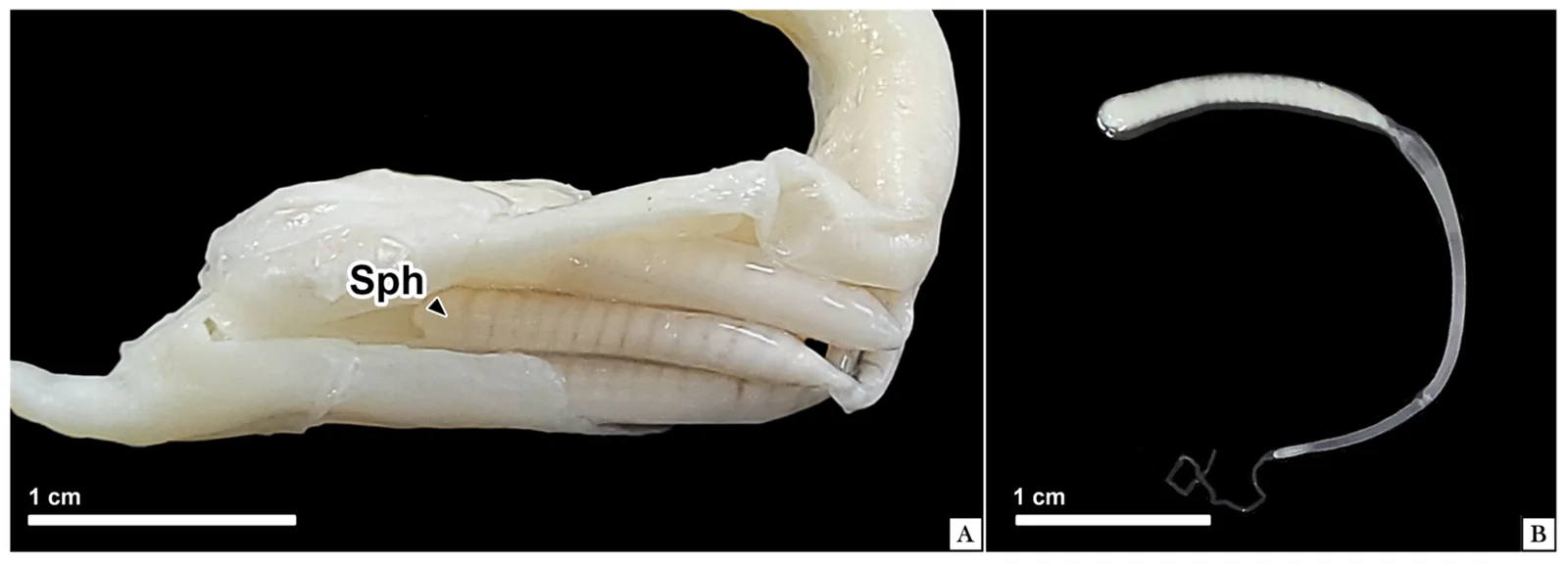
Spermatophore (Sph) in spermatophoric sac of male *Octopus minor*. (**A**) Spermatophores in spermatophoric sac and (**B**) spermatophore dissected from spermatophoric sac.

The presence or absence of spermatophores in the spermatophoric sac of the male was examined (Figure 3) and analyzed using the following equation.

(7) $$\mathrm{Frequency}\;\mathrm{of}\;\mathrm{male}\;\mathrm{with}\;\mathrm{spermatophore}\;(\%)\;=\;\frac{\mathrm{Males}\;\mathrm{with}\;\mathrm{spermatophores}\;(n)}{\mathrm{Male}\;(n)}\;\text{×}\;100$$

### 2.9. Sexual Group Maturity (SGM)

For the estimation of size at sexual group maturity, individuals that had reached or passed the onset of sexual maturity were grouped as sexually mature. Females were classified as sexually mature from the late active and mature stage, when mature oocytes began to appear, through the ripe stage, and the spent and degenerative stage. Males were classified as sexually mature from the late active and mature stage, when spermatophores were first observed in the spermatophoric sac, through the ripe stage, and the spent and degenerative stage.

Individuals were categorized into size groups at 10.0 g intervals for total weight (TW) and 5.0 mm intervals for mantle length (ML), and the size at 50% sexual maturity was estimated. A logistic regression model was applied to estimate the TW and ML at 50, 75, and 97.5% maturity.

### 2.10. Environmental Condition

Water temperature data for the sampling area were obtained from the Korea Hydrographic and Oceanographic Agency [35] to examine seasonal changes in water temperature together with the reproductive activity of *O. minor*.

### 2.11. Statistical Analysis

The sex ratio data were analyzed using a chi-square (χ^2^) test in IBM SPSS Statistics 29.0.2.0 software (IBM Corp., Armonk, NY, USA). A *p-*value < 0.05 was considered statistically significant. The maturity thresholds were estimated using a logistic model fitted with Solver in Microsoft Excel (Microsoft Corp., Redmond, WA, USA) and are reported as point estimates without confidence intervals.

## 3. Results

### 3.1. Sex Ratio

The overall sex ratio (female:male) was 1:1.03 (*n* = 159:163), and the proportion of females was 49.4%. The sex ratio according to TW tended to be male-biased from a group of 130.1–140.0 g TW, with a significant difference observed at 150.1 g TW and above (*p* < 0.05) (Table 1).

### 3.2. Gonadosomatic Index (GSI)

**Figure 4:**
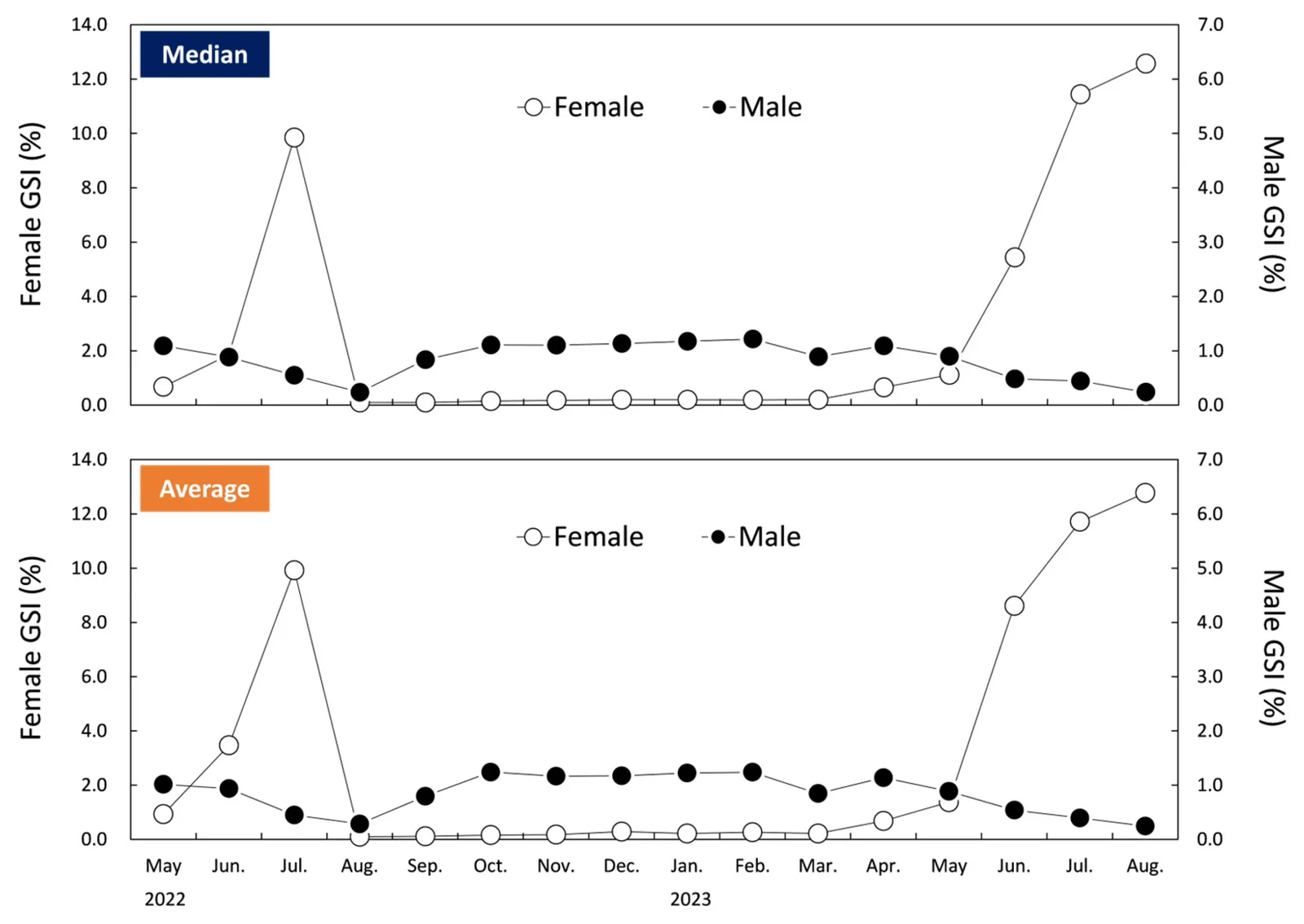
Monthly variation in gonadosomatic index (GSI) of *Octopus minor*.

In females, the GSI increased from May (median 0.68; average 0.93) to July (9.85; 9.92) 2022, and then sharply decreased in August (0.10; 0.10). Thereafter, the GSI remained low, at values below 0.30, until March 2023 (0.20; 0.22), and began to increase again from April (0.66; 0.68). It increased sharply in May (1.12; 1.37) and June (5.44; 8.61), followed by a continuous increase through August (12.58; 12.77) (Figure 4).

In males, the GSI remained below 1.50 throughout the study period. It began to decrease from May 2022 (median 1.09; average 1.01) and reached its lowest value in August (0.24; 0.29). Thereafter, the GSI increased again from September (0.84; 0.80) and remained around 1.10 until April 2023, before beginning to decrease again from May (0.90; 0.89) and reaching its lowest value in August (0.24; 0.24) (Figure 4).

### 3.3. Reproductive Complex Index (RCI)

**Figure 5:**
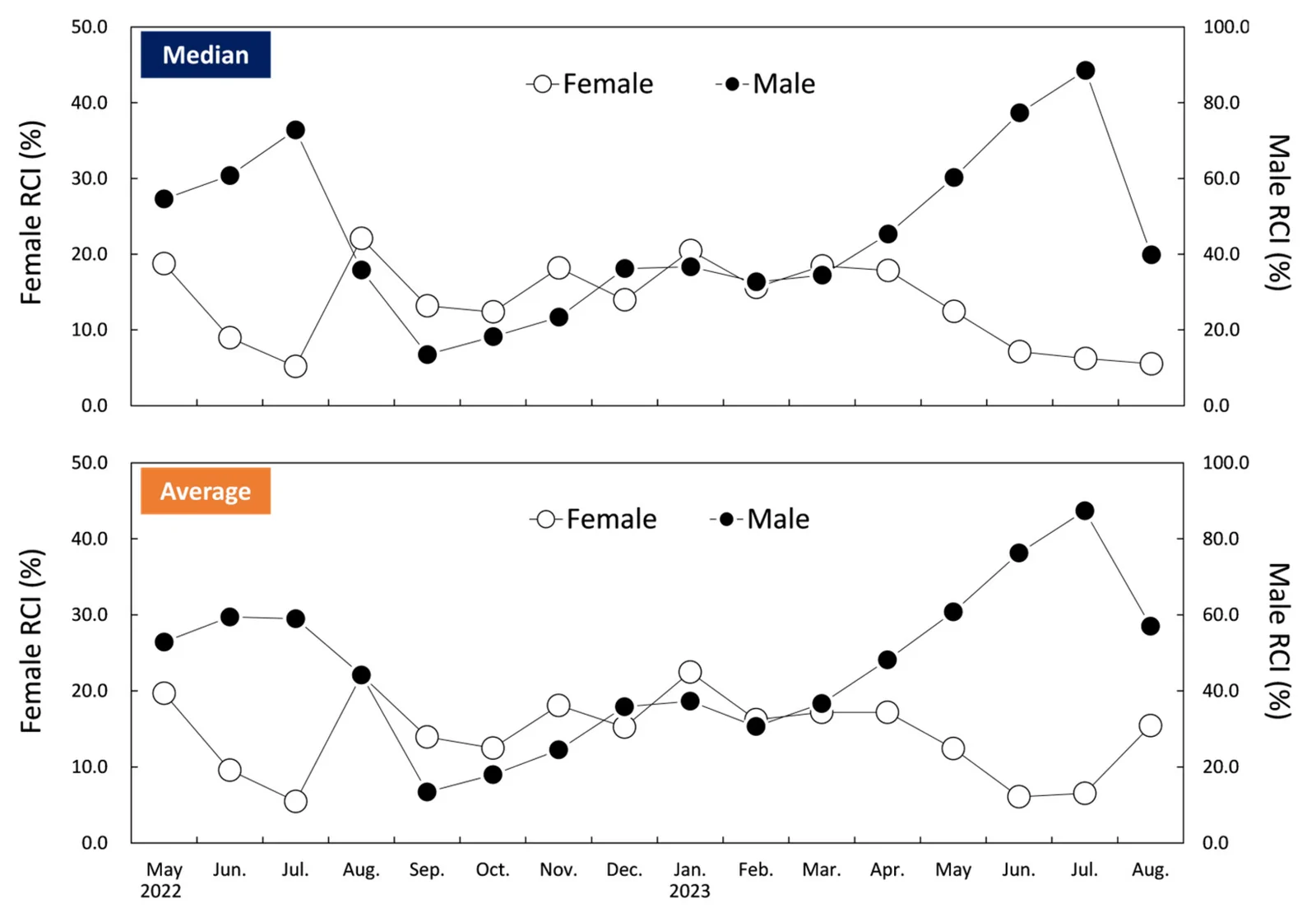
Monthly variation in reproductive complex index (RCI) of *Octopus minor*.

The female RCI decreased from May 2022 (median 18.75; average 19.70) and reached its lowest value in July (5.17; 5.49), followed by a sharp increase in August (22.09; 22.09). Thereafter, it fluctuated slightly between September and April, remaining around 15.0, and showed a decreasing trend from May (12.45; 12.43) (Figure 5).

The male RCI increased from May (median 54.57; average 52.86) to July (72.82; 58.98) 2022, followed by a decrease, reaching its lowest value in September (13.47; 13.43). Thereafter, it increased from October (18.25; 17.99) and reached its highest value in July 2023 (88.55; 87.40), followed by a sharp decrease (Figure 5).

### 3.4. Morphological and Histological Changes in the Gonads

#### 3.4.1. Female

**Figure 6:**
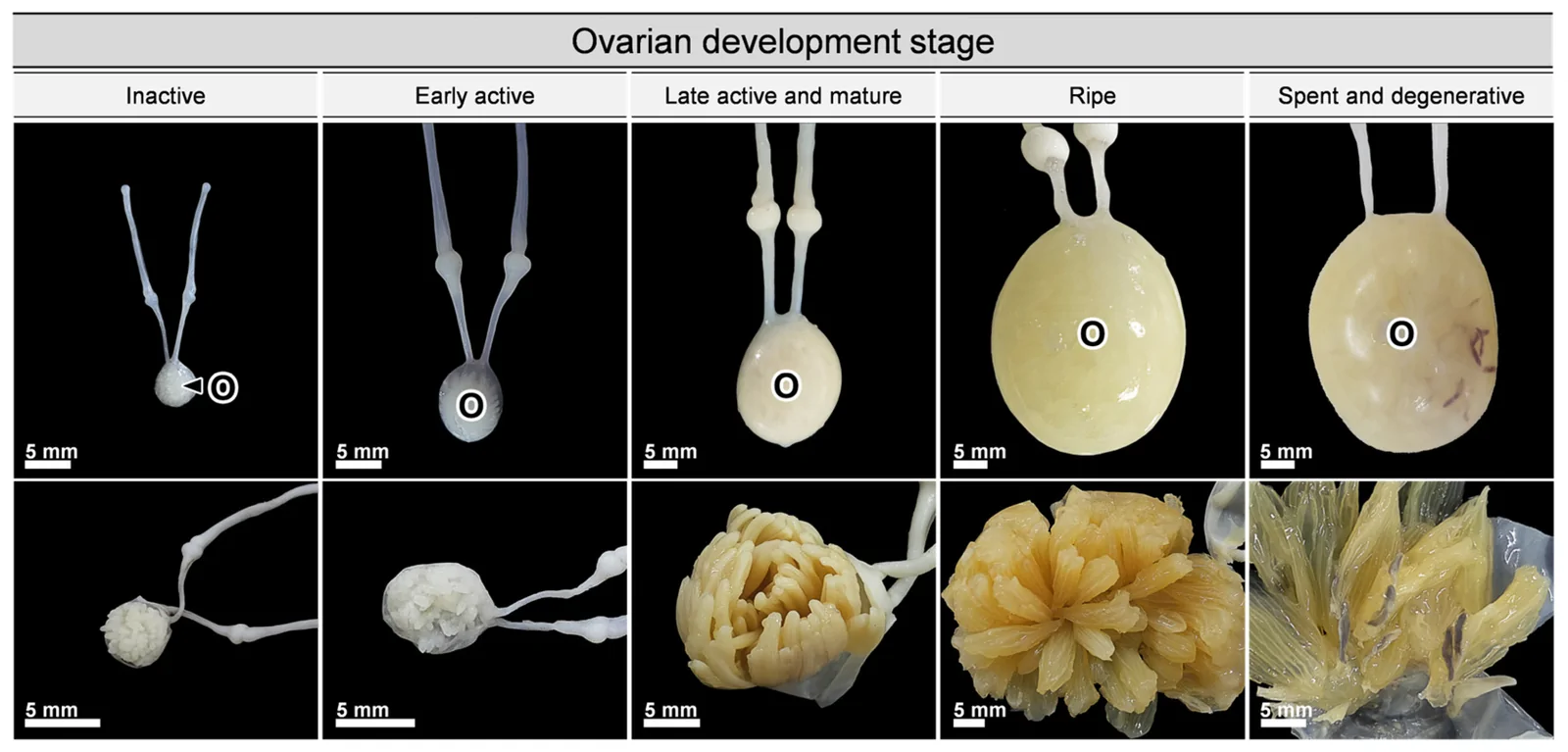
Morphological changes in the ovary (O) with ovarian development stage of *Octopus minor*.

**Figure 7:**
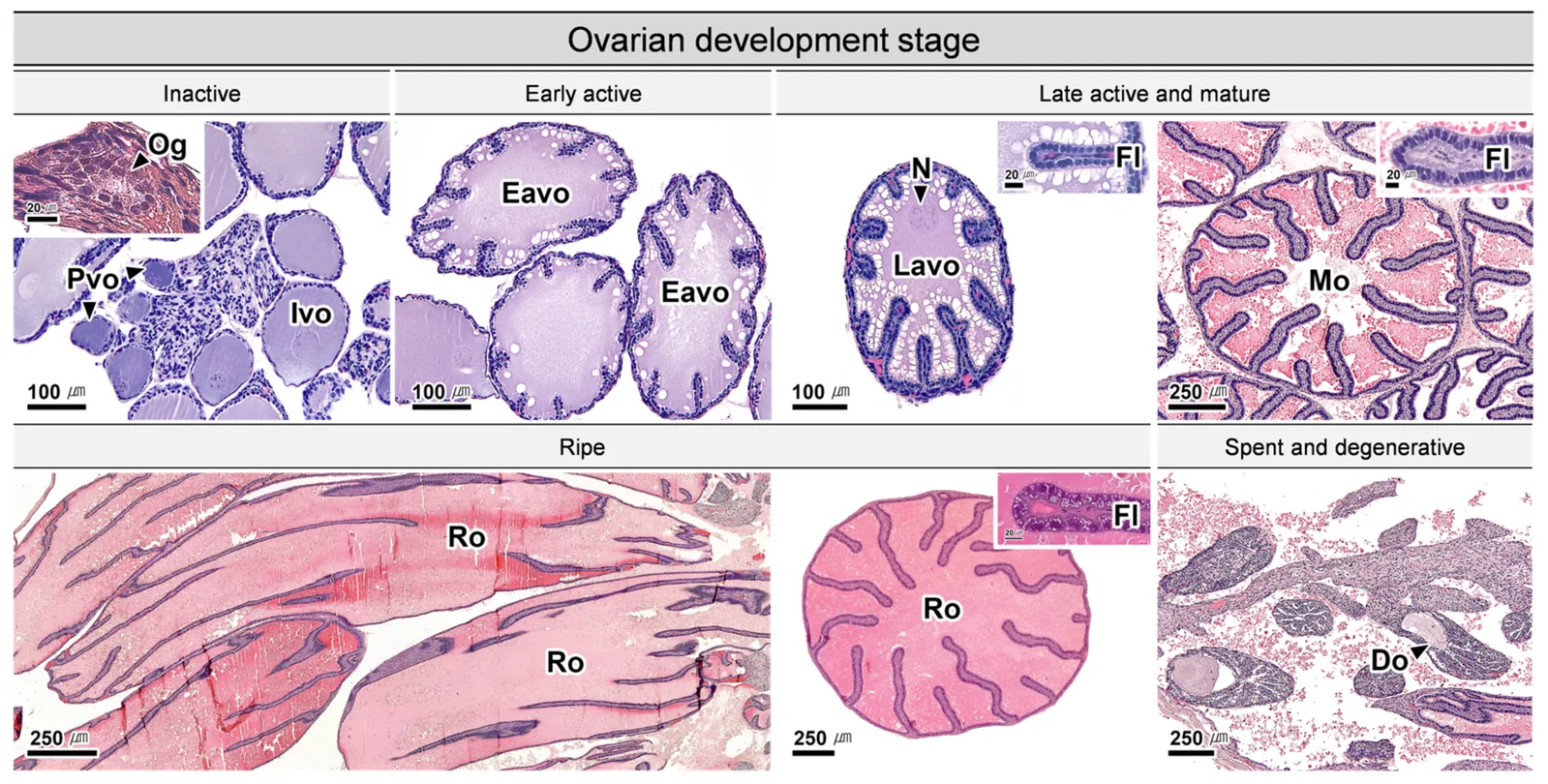
Histological changes in the ovary at different ovarian development stages of *Octopus minor*. Do: degenerative oocytes, Eavo: early active vitellogenic oocytes, Fl: follicle layer, Ivo: initial vitellogenic oocytes, Lavo: late vitellogenic oocytes, Mo: mature oocyte, N: nucleus, Og: oogonia, Pvo: previtellogenic oocytes, Ro: ripe oocytes.

In the inactive stage, the ovary was translucent and oval-shaped, measuring 6.35 (±0.52) × 5.00 (±0.71) mm (Figure 6). The predominant oocytes were oogonia (16.87 ± 7.19 × 11.19 ± 5.11 μm), previtellogenic oocytes (109.98 ± 22.48 × 75.56 ± 12.18 μm), and initial vitellogenic oocytes (345.17 ± 87.87 × 192.59 ± 58.81 μm) (Figure 7). Oogonia were clustered in the connective tissue of the ovary, and the basophilic nucleus occupied most of the cell. The cytoplasm of previtellogenic oocytes was basophilic, and the follicle layer was simple, with squamous follicle cells. Initial vitellogenic oocytes were translucent in appearance, and histologically, their cytoplasm was basophilic, similar to that of previtellogenic oocytes, while the follicle layer consisted of two layers (Figure 6 and Figure 7).

In the early active stage, the ovary was oval-shaped, measuring 7.24 (±1.02) × 5.65 (±1.01) mm, and showed a morphology similar to that of the inactive stage (Figure 6). The predominant oocytes at this stage were white and elongated in appearance, measuring 615.76 (±118.74) × 252.14 (±38.91) μm. Histologically, the cytoplasm of these oocytes was basophilic in H-E staining, with an increase in vesicular yolk granules, while the follicle layer showed well-developed folds, confirming the oocytes as early active vitellogenic oocytes (Figure 7).

In the late active and mature stage, the ovary was yellowish and oval-shaped, measuring 17.45 (±4.68) × 14.15 (±4.25) mm, approximately 2–3 times the size of that at the early active stage (Figure 6). The oocytes within the ovary were milky white and elongated, with a rectangular shape (Figure 6). The predominant oocytes in this stage were late active vitellogenic oocytes, measuring 1774.21 (±199.24) × 475.28 (±78.56) μm, and mature oocytes, measuring 8141.96 (±785.89) × 1485.73 (±182.19) μm (Figure 7). As late active vitellogenic oocytes developed into mature oocytes, the nucleus migrated toward the animal pole, the cytoplasm became eosinophilic, and the follicle cells became columnar (Figure 7).

In the ripe stage, the ovary was yellowish and oval-shaped, measuring 36.92 (±6.65) × 29.25 (±5.43) mm (Figure 6). The ovary was filled with ripe oocytes measuring 18,207.50 (±2394.42) × 4724.61 (±703.09) μm (Figure 7). The cytoplasm of these oocytes was filled with homogeneous eosinophilic yolk, and the eosinophilia of the follicle layer increased (Figure 7).

In the spent and degenerative stage, the ovary measured 42.75 (±5.61) × 34.33 (±4.84) mm and was morphologically similar to that at the ripe stage (Figure 6), but signs of spawning and oocytes measuring 4499.34 (±1622.88) × 990.00 (±248.81) μm were observed within the ovary (Figure 6 and Figure 7). Histologically, the cytoplasm of these oocytes was filled with eosinophilic yolk and basophilic granules, while the follicle layer showed an irregular shape, and the oocytes were identified as degenerative oocytes (Figure 7).

#### 3.4.2. Male

**Figure 8:**
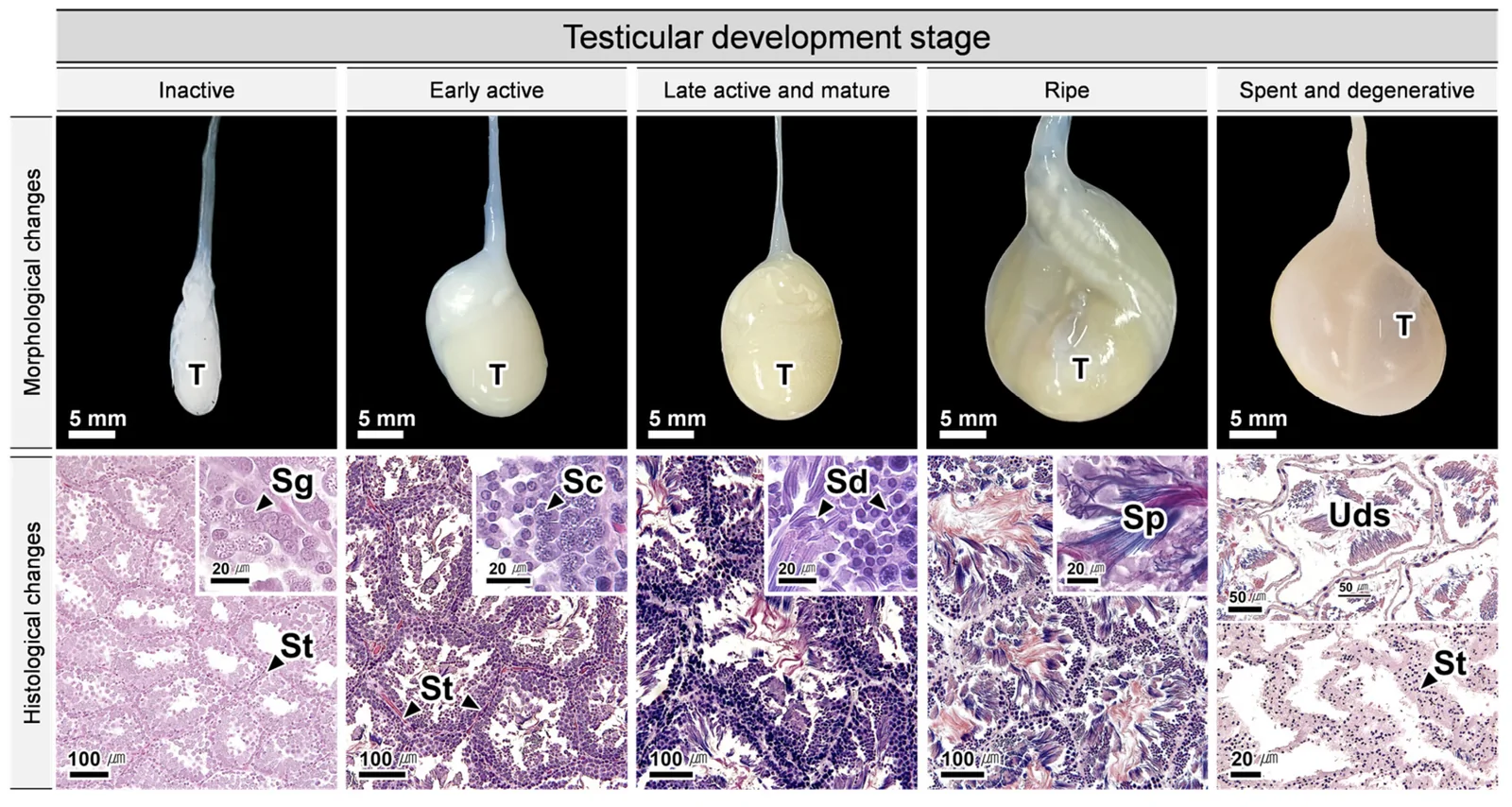
Morphological and histological changes in testis (T) with testicular development stage of *Octopus minor*. Sc: spermatocytes, Sd: spermatids, Sg: spermatogonia, Sp: sperm, St: seminiferous tubule, Uds: undischarged sperm.

In the inactive stage, the testis was oval-shaped and had a whitish appearance, measuring 4.56 (±0.71) × 3.22 (±0.54) mm (Figure 8). Histologically, spermatogonia, approximately 11.0 μm in diameter, were predominant along the periphery of the seminiferous tubules in the testis (Figure 8).

In the early active stage, the testis was pale milky white and oval-shaped, measuring 10.40 (±1.12) × 8.04 (±1.46) mm, and was approximately 2–3 times the size of that at the inactive stage (Figure 8). Histologically, enlargement of the lumen of the seminiferous tubules and the predominance of spermatocytes were observed. Spermatocytes were round, measuring 12.28 (±0.99) × 10.15 (±0.69) μm, with basophilic nuclei and weakly eosinophilic cytoplasm (Figure 8).

In the late active and mature stage, the testis was pale yellow and oval-shaped, measuring 14.73 (±2.15) × 12.10 (±2.81) mm (Figure 8). In the testis, spermatocytes, spermatids, and sperm showed a layered arrangement from the periphery toward the lumen of the seminiferous tubules, with spermatids being predominant. Spermatids were oval-shaped, with a more condensed nucleus than that of spermatocytes, and showed strong basophilic staining (Figure 8).

In the ripe stage, the testis was oval-shaped, measuring 17.48 (±2.43) × 14.47 (±1.71) mm, and increased in size by approximately 20% compared with that at the late active and mature stage (Figure 8). In the testis, some spermatocytes and spermatids were observed, while sperm were predominant in the lumen and showed strong basophilic staining (Figure 8).

In the spent and degenerative stage, the testis was pale brown and decreased in size to 12.37 (±2.59) × 10.56 (±2.79) mm compared with that at the ripe stage (Figure 8). In the testis, the seminiferous tubules became irregular in shape, and undischarged sperm were observed within the tubules (Figure 8).

### 3.5. Monthly Variation in Gonadal Developmental Stage

#### 3.5.1. Female

**Figure 9:**
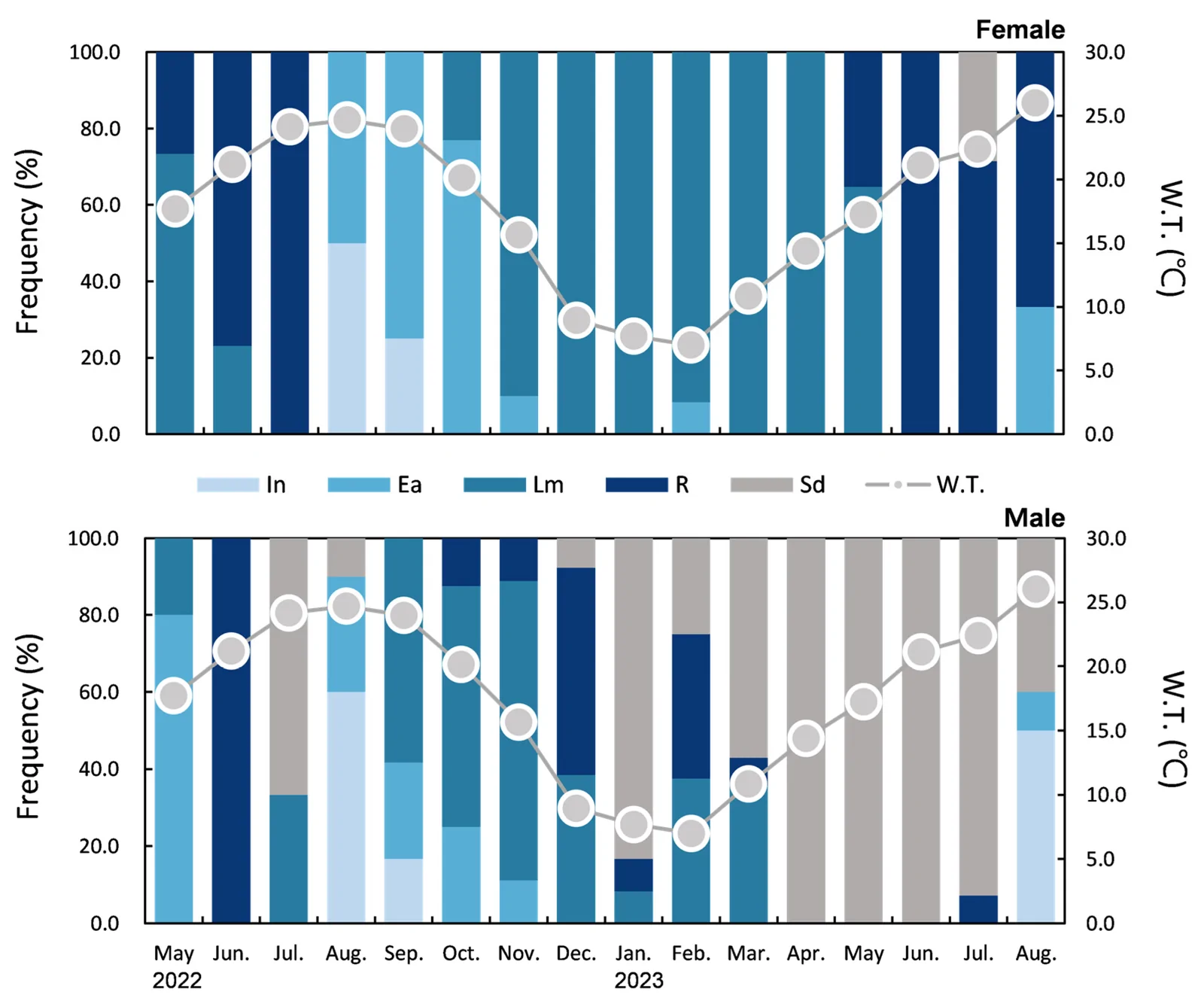
Monthly variation in gonadal developmental stage of *Octopus minor*. Ea: early active stage, In: inactive stage, Lm: late active and mature stage, R: ripe stage, Sd: spent and degenerative stage, W.T.: water temperature.

The inactive stage was mainly observed in August (50.0%) and September (25.0%) 2022, while the early active stage showed a high frequency from August to October 2022 (50.0–76.9%). The late active and mature stage showed a frequency of 73.3% in May 2022 and remained high from November 2022 to May 2023 (64.7–100.0%). The ripe stage showed a high frequency in June–July 2022 (76.9–100.0%) and June–August 2023 (66.7–100.0%), while the spent and degenerative stages showed a frequency of 28.6% in July 2023 (Figure 9).

#### 3.5.2. Male

The inactive stage was mainly observed in August (60.0%) and September (16.7%) 2022 and August 2023 (50.0%). The frequency of the early active stage was highest in May 2022 (80.0%) and ranged from 11.1 to 30.0% from August to November. The late active and mature stage was predominant from September to November 2022 (58.3–77.8%). The frequency of the ripe stage was 100.0% in June 2022 and ranged from 8.3 to 53.8% from October 2022 to February 2023. The spent and degenerative stage showed a frequency of 66.7% in July 2022 and ranged from 25.0 to 100.0% from January to August 2023 (Figure 9).

### 3.6. Monthly Frequency of Male with Spermatophore

**Figure 10:**
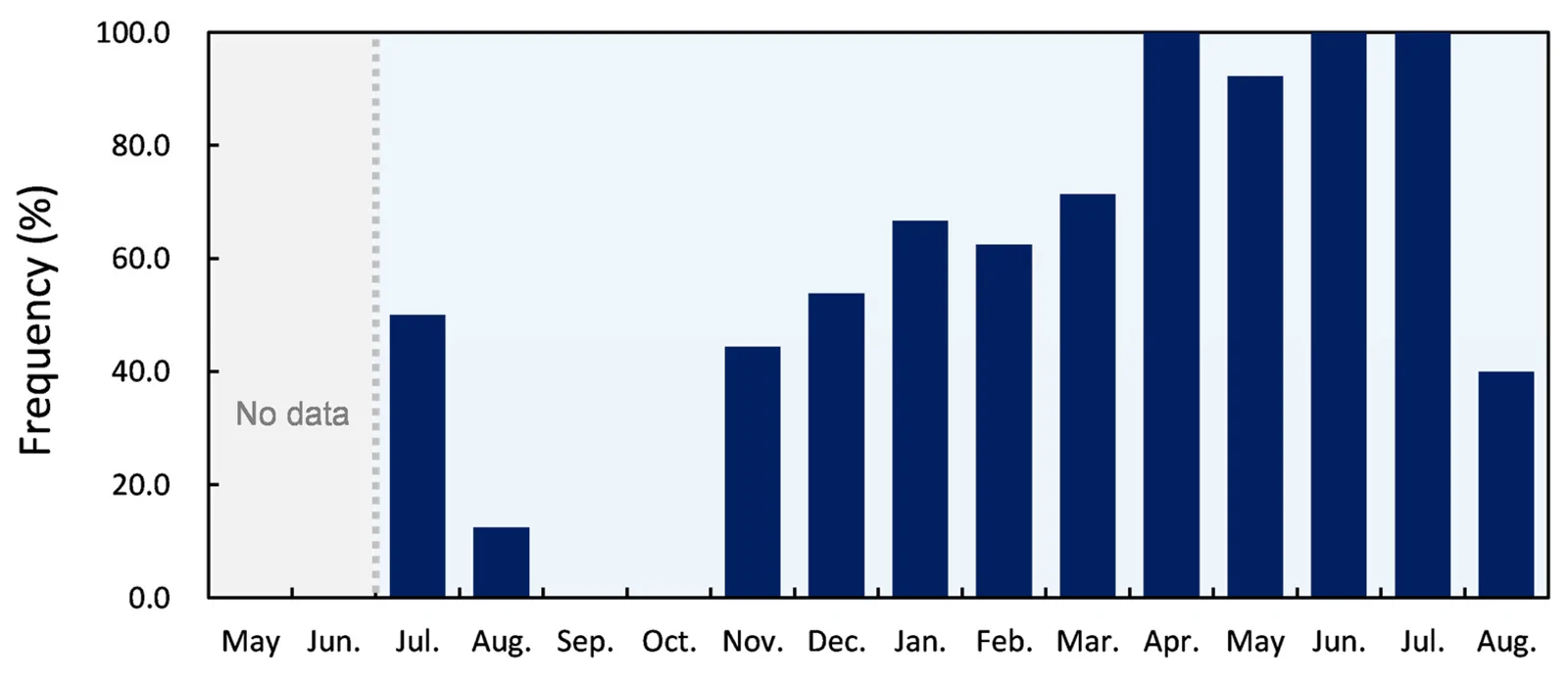
Monthly variation in the frequency of male with spermatophore of *Octopus minor*.

The monthly frequency of male with spermatophore decreased from July (50.0%) to October (0.0%) 2022, then continuously increased from November 2022 (44.4%) to April 2023 (100.0%), remained above 90.0% through July, and showed a decreasing trend again in August 2023 (40.0%) (Figure 10).

### 3.7. Sexual Group Maturity (SGM)

Female maturity was 83.3–100% in the group with 50.1 g TW or above and 45.5–100% in the group with 40.1 mm ML or above. Male maturity was 66.7–100% in groups with 50.1 g TW or greater and 68.8–100% in groups with 45.1 mm ML or greater (Table 2 and Table 3).

**Figure 11:**
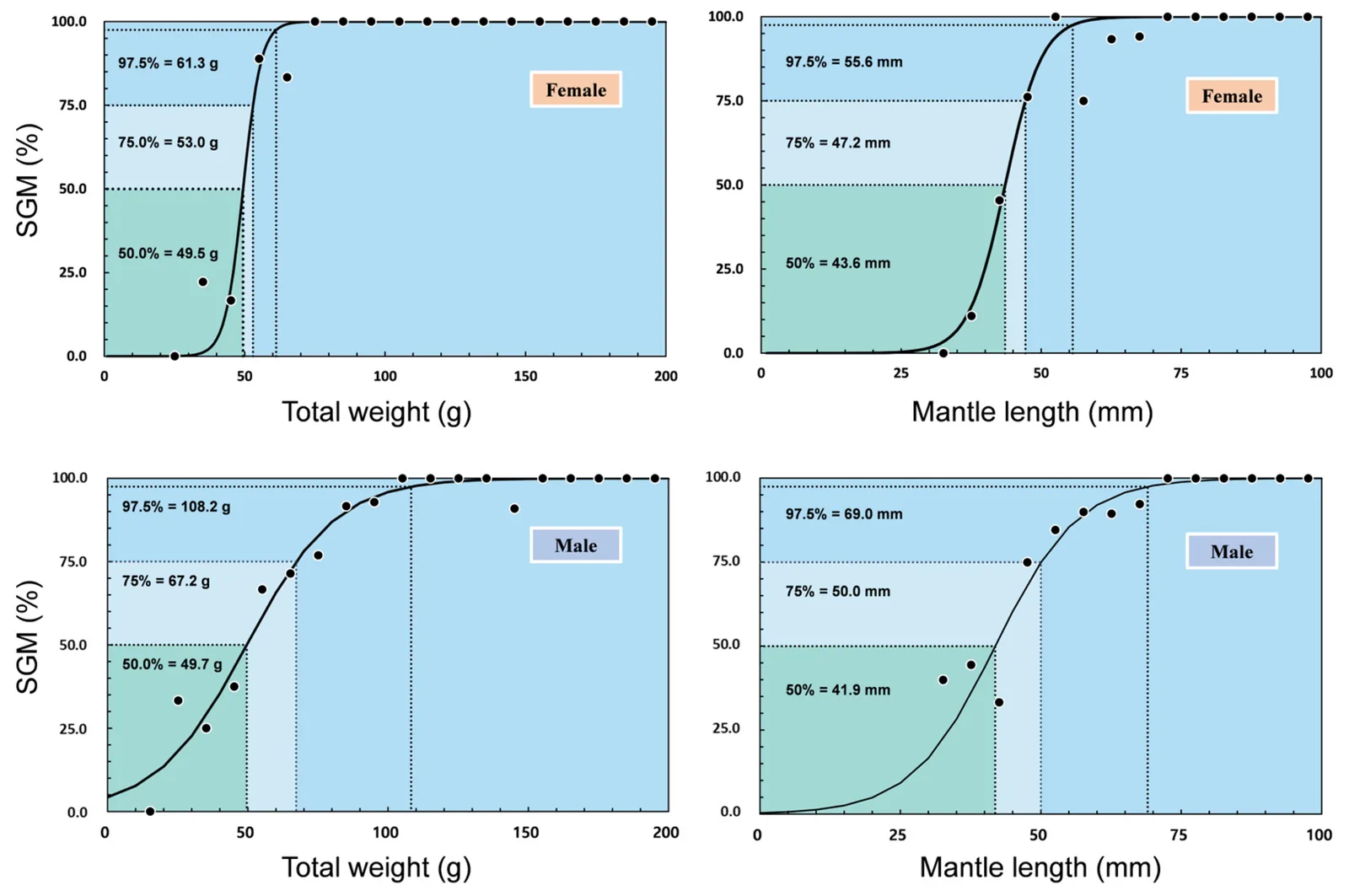
Relationship between size and sexual group maturity (SGM) of *Octopus minor*.

In females, the size at 50% SGM was estimated as 49.5 g TW and 43.6 mm ML; at 75% SGM, 53.0 g TW and 47.2 mm ML; and at 97.5% SGM, 61.3 g TW and 55.6 mm ML. In males, the size at 50% SGM was 49.7 g TW and 41.9 mm ML; at 75% SGM, 67.2 g TW and 50.0 mm ML; and at 97.5% SGM, 108.2 g TW and 69.0 mm ML (Figure 11).

**Table 2:** Sexual group maturity with total weight of *Octopus minor.*

Total Weight (g)	Maturity (%)			
	Female		Male	
≤30.0	0.0	(*n* = 0/7)	25.0	(*n* = 1/4)
30.1–40.0	22.2	(*n* = 2/9)	25.0	(*n* = 2/8)
40.1–50.0	16.7	(*n* = 2/12)	37.5	(*n* = 2/8)
50.1–60.0	88.9	(*n* = 8/9)	66.7	(*n* = 6/9)
60.1–70.0	83.3	(*n* = 10/12)	71.4	(*n* = 5/7)
70.1–80.0	100.0	(*n* = 13/13)	76.9	(*n* = 10/13)
80.1–90.0	100.0	(*n* = 16/16)	91.7	(*n* = 11/12)
90.1–100.0	100.0	(*n* = 13/13)	92.9	(*n* = 13/14)
100.1–110.0	100.0	(*n* = 17/17)	100.0	(*n* = 12/12)
110.1–120.0	100.0	(*n* = 7/7)	100.0	(*n* = 6/6)
120.1–130.0	100.0	(*n* = 12/12)	85.7	(*n* = 6/7)
130.1–140.0	100.0	(*n* = 7/7)	81.8	(*n* = 9/11)
140.1–150.0	100.0	(*n* = 5/5)	81.8	(*n* = 9/11)
150.1≤	100.0	(*n* = 20/20)	95.1	(*n* = 39/41)
Total	83.0	(*n* = 132/159)	81.0	(*n* = 132/163)

**Table 3:** Sexual group maturity with mantle length of *Octopus minor.*

Mantle Length (mm)	Maturity (%)			
	Female		Male	
≤40.0	10.0	(*n* = 1/10)	42.9	(*n* = 6/14)
40.1–45.0	45.5	(*n* = 5/11)	33.3	(*n* = 3/9)
45.1–50.0	76.2	(*n* = 16/21)	68.8	(*n* = 11/16)
50.1–55.0	100.0	(*n* = 17/17)	84.6	(*n* = 11/13)
55.1–60.0	75.0	(*n* = 15/20)	90.0	(*n* = 18/20)
60.1–65.0	93.3	(*n* = 14/15)	84.2	(*n* = 16/19)
65.1–70.0	94.1	(*n* = 16/17)	84.6	(*n* = 11/13)
70.1–75.0	100.0	(*n* = 15/15)	87.5	(*n* = 7/8)
75.1–80.0	100.0	(*n* = 9/9)	90.0	(*n* = 9/10)
80.1–85.0	100.0	(*n* = 1/1)	83.3	(*n* = 5/6)
85.1–90.0	100.0	(*n* = 7/7)	100.0	(*n* = 4/4)
90.1≤	100.0	(*n* = 16/16)	100.0	(*n* = 31/31)
Total	83.0	(*n* = 132/159)	81.0	(*n* = 132/163)

## 4. Discussion

Understanding sex ratio is important for assessing the reproductive characteristics of commercially important cephalopods. Sex ratio is an important indicator for predicting population size and differences in growth between sexes, but it may vary depending on population, maturity and spawning stage, sex-specific growth rates, and sampling methods [9,12,14,21]. Therefore, for accurate sex ratio analysis, analyzing the same number of specimens in each size group within the same population may reduce differences in the interpretation of the results [10].

Sex ratio has been reported to vary among populations of several species of the *Octopus* (Table 4). In Korea, the sex ratio of *Octopus minor* was reported as 1:0.96 in Cheonggye Bay [21], 1:1.15 in Garorim Bay [22], 1:0.85 in Deukryang Bay [23], and 1:1.05 in Tando Bay [24], with no significant differences between females and males. Similarly, the total sex ratio in the present study was 1:1.03, whereas the male ratio was significantly higher in the group of 150.1 g TW or above. Females of octopus [36,37] protect their eggs after spawning and undergo senescence, eventually dying after the eggs hatch. In contrast, males may continue to produce sperm and spermatophores and exhibit mating behavior during senescence until shortly before death [38,39]. In this study, the size at 50% sexual group maturity was similar between females (49.5 g TW) and males (49.7 g TW). Assuming that females protect their eggs and die after spawning at this size, the proportion of males would be expected to increase from approximately 50.0 g TW. However, this assumption does not adequately explain why a significant difference in the sex ratio was observed only in groups of 150.1 g TW or greater. Therefore, further studies on differences in growth between females and males and post-spawning mortality in *O. minor* are needed.

Changes in gonadosomatic index (GSI), along with changes in gonadal developmental stages, are important information for determining the rate of gonadal maturation and the main spawning season. The reproductive cycle of cephalopods is known to exhibit seasonal changes influenced by environmental factors, including photoperiod and water temperature [44], and this phenomenon has been reported in various *Octopus*, including *O. mimus* [45], *O. vulgaris* [10,15,40], and *O. maya* [18]. The GSI of *O. minor* females showed a seasonal change, increasing from May to July and then decreasing sharply after August. In contrast, the GSI of males decreased from May to August and then remained at high values from autumn to the following spring, showing a seasonal change different from that of females.

The reproductive complex of cephalopods consists of multiple organs that play important roles in reproduction, including spermatophore formation and internal fertilization. The reproductive complex index (RCI), which indicates the degree of development of the reproductive complex, has been used to investigate the reproduction of Octopodidae [13,14,15,16,32,40].

The RCI of *O. minor* showed a pattern opposite to that of GSI. The RCI of females decreased during summer and then increased again in autumn, showing a trend opposite to that of GSI. These results have also been reported in *O. vulgaris* [12,13,16], *O. insularis* [42], and *O. hubbsorum* [20], which can be interpreted as a result of the more rapid increase in ovary weight than in oviducal gland weight during gonadal development [16,20].

The RCI of males increased from spring to summer, showing a trend opposite to that of GSI, then decreased in autumn and subsequently increased continuously from autumn to the following summer. This seasonal trend in RCI was similar to those reported for *O. vulgaris* [13,14,16], *O. insularis* [42], and *O. hubbsorum* [20]. The opposing patterns of change in GSI and RCI of males may be associated with the transfer of sperm from the testis to the reproductive complex, where sperm are subsequently incorporated into spermatophores. Thus, the increase in RCI may reflect increased activity of the reproductive complex associated with spermatophore formation and accumulation.

GSI is calculated directly from gonad weight and has been widely used to assess gonadal development and reproductive activity in cephalopods, with most studies using average values to describe monthly variations [15,20,42]. Because GSI is based on individual gonad weight, monthly average values may be substantially affected by a few specimens with extremely low or high values, particularly when the sample size is small. Shin et al. [10] reported that the GSI of female largehead hairtail, *Trichiurus japonicus*, showed a difference between the median and average values and recommended using the median together with the average. Therefore, in the present study, both median and average values were used to describe monthly variations in GSI.

In this study, the GSI and RCI of *O. minor* showed similar monthly variation patterns between the median and average values. However, considerable differences between the median and average values were observed for female GSI in June 2022 (median 1.85; average 3.47) and June 2023 (5.44; 8.61), female RCI in August 2023 (5.53; 15.45), and male RCI in July (72.82; 58.98) and August 2022 (35.83; 44.21) and August 2023 (39.84; 57.06). Considering these differences, the use of both median and average values is recommended.

The gonadal developmental stages of marine animals are quantified through macroscopic and microscopic analyses and used as important data for determining the reproductive cycle, main spawning season, and sexual group maturity. However, gonadal developmental stages may differ among researchers, even in studies of the same species [14,15,16,20,28].

A comparison of the gonadal developmental stages of *O. vulgaris* based on macroscopic and microscopic analyses showed that two or more stages identified by macroscopic analysis were classified as the same stage by microscopic analysis [28]. The testicular developmental stages were classified into four stages by Silva et al. [14] and Otero et al. [15] based on GSI, RCI, and macroscopic analysis, whereas Cuccu et al. [16] classified them into six stages based on macroscopic and microscopic analyses. The ovarian developmental stages were classified into five stages by Otero et al. [15] based on GSI, RCI, and macroscopic analysis, whereas Alejo-Plata and Gómez-Márquez [20] classified them into six stages based on microscopic analysis. In *O. minor*, Kim and Kim [21] classified the gonadal developmental stages as immature, mature, and ripe based on the morphology and size of oocytes in the ovary. However, in the present study, gonadal developmental stages were classified as inactive, early active, late active and mature, ripe, spent and degenerative based on both macroscopic and microscopic analyses of the gonads. Thus, although macroscopic analysis of gonadal developmental stages can save time and cost compared with microscopic analysis, it may lead to errors in interpretation, highlighting the importance of histological analysis in validating morphological classification of gonadal developmental stages. Monthly variation in the gonadal developmental stage is one of the reproductive indicators used to understand the maturation process and duration of gonadal development. Gonadal development in cephalopods is known to be influenced by various environmental factors, including latitude, water temperature, photoperiod, and food availability [34]. In *O. vulgaris*, females matured in May and August, whereas males matured throughout the year in the Gulf of Cádiz [14], and females matured in August and September, whereas males matured throughout the year along the Atlantic coast of Andalusia [41]. In *O. maya*, females matured from February to June, whereas males matured in March and from May to June along the coast of Sisal [18]. In *O. hubbsorum*, females matured in April–May and October–November, whereas males matured throughout the year along the coast of Oaxaca [20], while females matured in January and December and males matured throughout the year in the Gulf of California [46]. In the present study, monthly variation in the gonadal developmental stage of *O. minor* showed seasonal variation patterns, although the patterns differed between females and males, consistent with previous studies [14,18,20,41,46].

In reproduction studies of internal-fertilization cephalopods, reports on the mating season and sperm storage period in females after mating are scarce. The RCI, which reflects the activity of organs related to spermatophore formation in male *O. minor*, and the monthly frequency of male with spermatophore decreased sharply during summer (July–August), suggesting that the main mating season occurs in summer. However, these results were indirectly estimated based on the male reproductive index. Therefore, further studies on mating behavior and the sperm storage rate in the female oviducal gland are needed.

Information on the main spawning season is important for studies of the reproductive ecology of the species and the establishment of a prohibited fishing season, and the spawning season varies depending on the region and environmental factors such as water temperature [10]. The spawning season of *O. vulgaris* has been reported as April–May and August–September in the Gulf of Cádiz [14], April and October–November along the coast of the Canary Islands [40], February–April and June–July along the northwest coast of the Portuguese coastal area, and August–September along the south coast [47]. The spawning season of *O. hubbsorum* has been reported as April–May and October–November along the coast of Oaxaca [20], while that of *O. maya* has been reported as February–June along the coast of Sisal [18]. The main spawning season of *O. minor* in Deukryang Bay, Korea, was July–August, showing regional differences as well. In addition, differences in the monthly occurrence of gonadal developmental stages were observed between 2022 and 2023. The reproductive cycle and spawning season of *Octopus* may vary depending on environmental conditions [47]. However, based on the water temperature data in the present study, it was difficult to determine whether water temperature affected the differences in the monthly occurrence of gonadal developmental stages between 2022 and 2023. Therefore, long-term studies may be necessary to better understand the annual variation in the reproductive activity of *O. minor*.

The oocyte development pattern provides important information on the spawning pattern and number of spawning events. Wallace and Selman [48] classified the oocyte development patterns of teleosts into synchronous, group-synchronous, and asynchronous patterns based on the distribution of predominant oocytes in the ovary.

Understanding the reproductive strategy of a species is essential for elucidating its lifecycle traits and reproductive ecology and for effective resource management and conservation. Cole [49] classified the reproductive patterns of organisms into semelparity, in which spawning occurs only once during the lifetime, and iteroparity, in which spawning occurs two or more times, based on the number of spawning events. Mangold [34] classified the spawning types of cephalopods into four categories based on the number and timing of spawning events during the annual reproductive cycle: (1) strict seasonality, in which spawning occurs only during a specific season; (2) extended seasonality, in which spawning occurs across multiple seasons; (3) year-round seasonality, in which spawning occurs throughout the year; and (4) two spawning periods within one year, in which spawning occurs twice a year. Subsequently, Rocha et al. [50] further subdivided semelparity and iteroparity by considering the oocyte development pattern and spawning pattern, and classified the reproductive strategies of cephalopods into five types. (1) Simultaneous terminal spawning is characterized by a synchronous pattern and a monocyclic spawning pattern. This type is semelparous, with a single spawning event occurring over a very short period at the end of the lifetime, and includes *O. vulgaris* [34,42], *O. cyanea* [51], *Todarodes pacificus* [52], and *O. mimus* [45]. (2) Polycyclic spawning is characterized by a group-synchronous pattern and a polycyclic spawning pattern. This type is characterized by the regeneration of the ovary following spawning, allowing repeated spawning over multiple spawning seasons, and includes *Nautilus* spp. [53,54]. (3) Multiple spawning is characterized by a group-synchronous pattern and a monocyclic spawning pattern. This type is characterized by multiple spawning events within a single spawning season, and *O. chierchiae* [55] belongs to this type. (4) Intermittent terminal spawning is characterized by a group-synchronous pattern and a monocyclic spawning pattern. According to the classification of Rocha et al. [50], this type differs from multiple spawning in that somatic growth does not occur between spawning seasons and is mainly observed in squids and cuttlefish, including *Sepia officinalis* [56], *Loligo vulgaris* and *L. forbesi* [57], and *Illex coindetii* [58]. (5) Continuous spawning is characterized by an asynchronous pattern and a monocyclic spawning pattern. This type has been reported in *Cirrothauma murrayi* [59] and *Opisthoteuthis agassizii* [60].

In the present study, microscopic analysis showed that the morphology and size of oocytes in *O. minor* were mostly similar, while the monthly variation in GSI showed a single peak in summer. Therefore, the oocyte development pattern of *O. minor* was identified as a synchronous pattern, and the main spawning season was identified as summer (July–August), indicating a monocyclic spawning pattern with strict seasonality. These results indicate that the reproductive strategy of *O. minor* is simultaneous terminal spawning.

Information on the size at 50% sexual group maturity is important for resource conservation and the establishment of a size limit. The criteria for determining maturity in group maturity analyses vary among researchers, even within the same species, and particularly when using histological methods, errors may occur in the interpretation of the results [29,30]. Such cases have been reported in *Saxidomus purpuratus* [26] and *T. japonicus* [10].

Reports on the size at 50% sexual group maturity of *O. minor* also differed among researchers. Kim and Kim [21] analyzed the morphology of the gonad and eggs macroscopically and classified gonadal development into three stages, immature, mature, and ripe, considering individuals at the mature and ripe stages as sexually mature, and reported the size at 50% sexual group maturity as 70.6 mm ML. Kim et al. [22] classified gonadal development into six stages using a histological method and considered individuals at or beyond the mature stage as sexually mature, reporting a size at 50% sexual group maturity of 35.8 mm ML for females and 55.6 mm ML for males. Additionally, Lee et al. [23] and Lee et al. [24] identified sexually mature individuals based on macroscopic observations and reported sizes of 102.3 g TW and 158.0 g TW, respectively, which differed considerably from the results of the present study, with 49.5 g TW and 43.6 mm ML for females and 49.7 g TW and 41.9 mm ML for males.

These differences may be attributed to differences in the classification of gonadal development stages and the criteria used to identify sexually mature individuals. In particular, histological analysis of the reproductive complex is needed for males. In the present study, females at the late active and mature, ripe, spent, and degenerative stages were considered sexually mature individuals. In males, spermatophores were first observed in the spermatophoric sac at the late active and mature stage; therefore, individuals at the late active and mature, ripe, spent, and degenerative stages were considered sexually mature.

The information obtained in this study regarding the main spawning season (July–August) and size at 50% sexual group maturity (F: TW 49.5 g, ML 43.6 mm/M: TW 49.7 g, ML 41.9 mm) of the *O. minor* population in Deukryang Bay, Korea provides biological baseline data that may contribute to future fisheries management, including the establishment of a prohibited fishing season and size limit. However, because the reproductive characteristics of *O. minor* may vary among regions and populations, regional reproductive characteristics and resource ecological information should also be considered when establishing specific management measures.

## 5. Conclusions

This study analyzed the sex ratio, GSI, RCI, and gonadal development stage of *Octopus minor* in Deukryang Bay, Korea, to understand its reproduction and provide basic information for resource management. The sex ratio (female:male) was 1:1.03, with no significant difference, although the proportion of males tended to be higher from group of 130.1 g TW and showed a significant difference at 150.1 g TW and above. Therefore, further studies on differences in growth between females and males and post-spawning mortality in *O. minor* are needed. The temporal dynamics of reproductive activity showed seasonal variation, and the main mating and spawning season was estimated to be summer (July–August). The oocyte development pattern was synchronous, while the spawning pattern was monocyclic with strict seasonality, indicating that the reproductive strategy of *O. minor* is simultaneous terminal spawning. The size at 50% sexual group maturity was 49.5 g TW and 43.6 mm ML in females, and 49.7 g TW and 41.9 mm ML in males. The differences between the results of the present study and previous reports were interpreted as being due to differences in the classification of gonadal developmental stages and the criteria for sexual maturity. The information on the main spawning season and size at 50% sexual group maturity can be considered for the establishment of a prohibited fishing season and size limit for resource management of *O. minor* in Deukryang Bay, Korea.

## Figures and Tables

**Table 1:** Sex ratio with total weight of *Octopus minor.*

Total Weight (g)	Number			Sex Ratio (F:M)	Female (%)	Chi-Square	*p*-Value
	Total	Female	Male				
≤30.0	11	7	4	1:0.57	63.6	0.818	0.366
30.1–40.0	17	9	8	1:0.89	52.9	0.059	0.808
40.1–50.0	20	12	8	1:0.67	60.0	0.800	0.371
50.1–60.0	18	9	9	1:1.00	50.0	-	1.000
60.1–70.0	19	12	7	1:0.58	63.2	1.316	0.251
70.1–80.0	26	13	13	1:1.00	50.0	-	1.000
80.1–90.0	28	16	12	1:0.75	57.1	0.571	0.450
90.1–100.0	27	13	14	1:1.08	48.1	0.037	0.847
100.1–110.0	29	17	12	1:0.71	58.6	0.862	0.353
110.1–120.0	13	7	6	1:0.86	53.8	0.077	0.782
120.1–130.0	19	12	7	1:0.58	63.2	1.316	0.251
130.1–140.0	18	7	11	1:1.57	38.9	0.889	0.346
140.1–150.0	16	5	11	1:2.20	31.3	2.250	0.134
150.1≤	61	20	41	1:2.05	32.8	7.230	0.007
Total/Mean	322	159	163	1:1.03	49.4	0.050	0.824

F: female, M: male.

**Table 4:** Previous studies of sex ratio in *Octopus* sp.

Species	Sampling Area	Sex Ratio (F:M)	Citation
*O. vulgaris*	Gulf of Cádiz	1:0.94	Silva et al., 2002 [14]
	Canary Island	1:1.95	Hernández-García et al., 2002 [40]
	Huelva, Southwest of Spain	1:1.10	Rodríguez-Rúa et al., 2005 [41]
	Galician water, Spain	1:0.88	Otero et al., 2007 [15]
*O. hubbsorum*	Central Mexican Pacific	1:1.15	López-Uriate and Ríos-Jara, 2009 [19]
	Oaxaca coast, Mexico	1:0.76	Alejo-Plata and Gómez-Marquez, 2015 [20]
*O. insularis*	Rio do Fogo coast, Brazil	1:0.89	de Lima et al., 2014 [42]
	Veracruz Reef System, Mexico	1:1.11	González-Gómez et al., 2020 [43]
*O. maya*	Sisal coast, Yucatan, Mexico	1:1.41	Avila-Poveda et al., 2016 [18]
*O. minor*	Cheonggye Bay, Southwest coast of Korea	1:0.96	Kim and Kim, 2006 [21]
	Garorim Bay, West coast of Korea	1:1.15	Kim et al., 2017 [22]
	Deukryang Bay, Korea	1:0.85	Lee et al., 2017 [23]
	Tando Bay, Southwest coast of Korea	1:1.05	Lee et al., 2018 [24]
	Deukryang Bay, Korea	1:1.03	Present study

F: female, M: male.

## Data Availability

The original contributions presented in this study are included in the article. Further inquiries can be directed to the corresponding authors.
